# Superior mesenteric vein thrombosis due to COVID-19 vaccination: a case report

**DOI:** 10.1186/s13256-023-04320-2

**Published:** 2024-01-11

**Authors:** Keita Suto, Akira Saito, Katsusuke Mori, Atsushi Yoshida, Naohiro Sata

**Affiliations:** 1 Department of Surgery, Koga Red Cross Hospital, 1150 Shimoyama-Cho, Koga-Shi, Ibaraki, 306-0014 Japan; 2 Department of Surgery, Division of Gastroenterological, General and Transplant Surgery, Jichi Medical University, 3311-1 Yakushiji, Shimotsuke-Shi, Tochigi, 329-0498 Japan

**Keywords:** Case report, COVID-19 vaccination, mRNA-1273, Mesenteric ischemia, VITT

## Abstract

**Background:**

The worldwide vaccination response to COVID-19 has been associated with rare thrombotic complications, including the case of postvaccination splanchnic venous thrombosis we report here.

**Case presentation:**

An 80-year-old Japanese male with abdominal pain presented to our hospital six days after receiving a dose of the COVID-19 messenger ribonucleic acid vaccine. Abdominal computed tomography showed localized edema of the small intestine, increased density of the surrounding adipose tissue, and a thrombus in the superior mesenteric vein. Conservative inpatient treatment with unfractionated heparin relieved the thrombosis, and the patient is currently receiving oral apixaban as an outpatient.

**Conclusion:**

Reported cases of thrombosis after COVID-19 vaccination typically have been associated with viral vector vaccines, with few reports of thrombosis induced by mRNA vaccines. The potential for venous thrombosis should be explored when patients present with abdominal pain soon after COVID-19 vaccination.

## Background

The virus causing the severe acute respiratory disease COVID-19 has infected 762.2 million people worldwide and killed 6.89 million people (as of 5 April 2023) [1]. To avert this global emergency, vaccines have made a significant contribution to slowing the spread of the infection. However, side effects of COVID-19 vaccination are beginning to appear, one of which, thrombosis, is a particular problem because it can cause serious complications. Among thrombosis, splanchnic venous thrombosis is closely related to intestinal ischemia. Although there are some reports of splanchnic venous thrombosis (SVT) after vaccination with ChAdOx1 nCoV-19 vaccines, but case reports of SVT after vaccination with mRNA-1273 vaccines are rare. This report examines the relationship between messenger ribonucleic acid (mRNA)-1273 vaccines and intestinal ischemia through this case. It is important for clinicians to consider the possibility of vaccine-related SVT when examining patients with abdominal pain after receiving the COVID-19 vaccine. Here, we report our experience with a patient who presented with SVT after receiving a dose of the COVID-19 vaccine.

## Case presentation

An 80-year-old Japanese male presented to our hospital with abdominal pain.

**Fig. 1:**
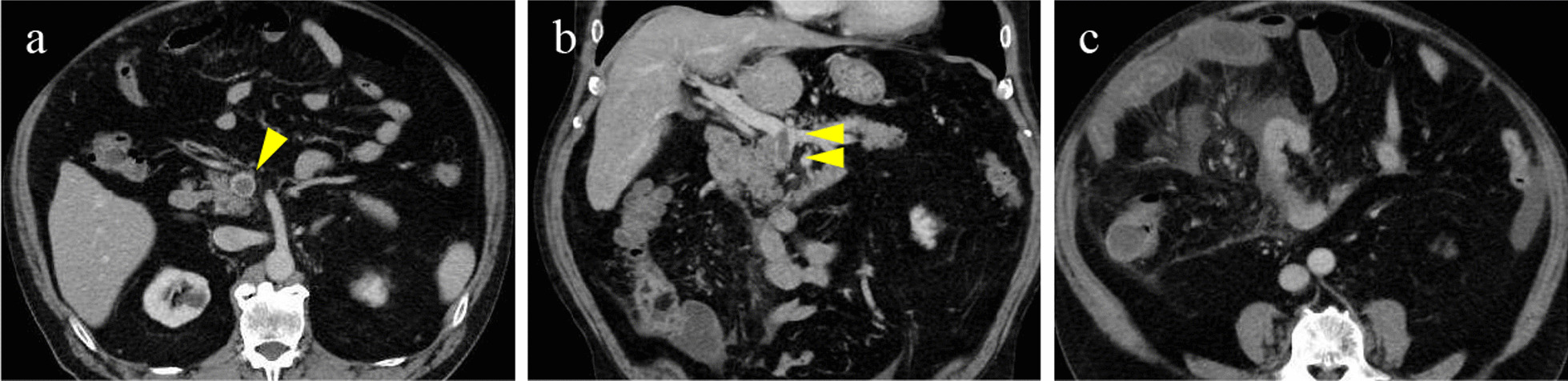
Abdominal computed tomography on admission. Yellow arrows: Thrombosis in the superior mesenteric vein. Axial image (**a**). Coronal image (**b**). Increased adipose tissue density in the mesentery of the small intestine, focal edema of the small intestine, and ascites around the small intestine (**c**)

His past medical history was hypertension, hyperuricemia, and prostate cancer. There was no particular family history. His medication included tablet nifedipine 10 mg, Allopurinol 100 mg and Tamsulosin Hydrochloride 0.2 mg orally twice daily. He was not a smoker and drinks socially. His prostate cancer was diagnosed at the urology department of our hospital 4 years before he visited our department. The stage of prostate cancer was cT2aN0M0, the Gleason score was 4 + 5, and the PSA at the time of diagnosis was 4.8 ng/mL (0–4.0). Although the Gleason score was high, active surveillance was performed without medication treatment because the tumor was localized. He had received a dose of the mRNA-1273 COVID-19 vaccine (Moderna) 6 days prior to presentation. He had received the BNT162b2 mRNA vaccine (BioNTech/Pfizer) four times in the past, and the only side effect after vaccination was fever, which improved within a few days. This was the first time he had received the mRNA-1273 COVID-19 vaccine. On presentation, his vital signs were a temperature of 36.5 °C, blood pressure of 128/72 mmHg and heart rate of 58 beats per minute and he was obese (height, 160.2 cm; weight, 85.6 kg; body mass index, 33.05 kg/m^2^). Physical examination revealed epigastric tenderness with no evidence of peritonism. No abnormalities were observed in respiratory sounds or heart sounds. He had no obvious neurological abnormalities. Results (reference range) of laboratory tests at presentation were: white blood cell count, 10,400 cells/μl (3300–8800); hemoglobin, 16.9 g/dl (14.0–18.0); hematocrit, 50.0% (36.5–55.3); mean corpuscular volume, 102.2 fl (85–104); platelet count, 11.3 × 10^4^/μl (12–28); C-reactive protein, 9.55 mg/dl (0–0.3); prothrombin international normalized ratio, 0.93 (0.85–1.15); activated prothrombin time, 29.5 s (20–40); D-dimers, 19.8 µg/ml (0–1.0); and fibrinogen, 539 mg/dl (162–358); aspartate aminotransferase, 24 IU/L (10–33); alanine aminotransferase, 23 IU/L (5–33); lactate dehydrogenase, 230 IU/L (89–231);γ-glutamyltranspeptidase, 50 IU/L (10–87); total bilirubin, 1.0 mg/dL (0.2–1.2); blood urea nitrogen, 25.0 mg/dL (8–20); creatinine, 1.22 mg/dL (0.6–1.11); sodium, 146.2 mEq/L (136–147); potassium, 4.9 mEq/L (3.6–5.0); chlorine, 111.4 mEq/L (98–109). His urine test revealed no obvious abnormalities. No bacteria were detected in blood culture. Abdominal Computed Tomography (CT) showed thrombosis centering on the superior mesenteric vein (SMV) and extending to the portal vein, increased adipose tissue density in the mesentery of the small intestine, and localized edema of the small intestine. Ascites was present around the edematous small intestine, but intestinal contrast enhancement was preserved (Fig. 1).

Results of additional laboratory tests ruled out other congenital or acquired risk factors for thrombus formation in our patient: protein S, 133% of control; protein C, 120%; anti-cardiolipin IgG antibody, < 4.0 U/ml; anti-cardiolipin β2GPI antibody, < 1.2 U/mL (< 3.49); and lupus anticoagulant, 45.4 s (< 46.5). In addition, anti-platelet factor 4 (PF-4) antibody testing (5 U/ml) was negative. Given these combined results, our patient was diagnosed with SVT, specifically SMV thrombosis, and was hospitalized. COVID-19 reverse transcription-polymerase chain reaction (RT-PCR) analysis at the time of hospitalization was negative. He received ceftriaxone at dosage of 1 g IV every 12 hours on fasting and was started on 10,000 IU of unfractionated heparin daily.

**Fig. 2:**
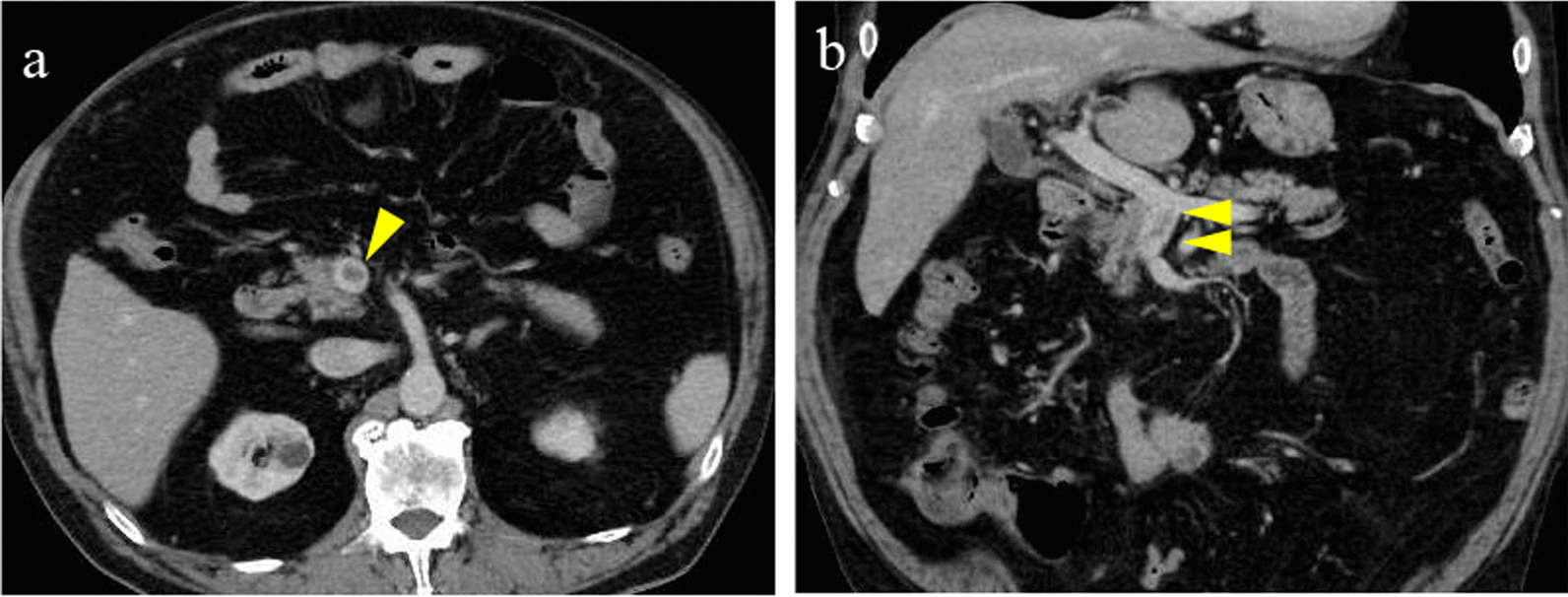
Abdominal computed tomography at 12 days after starting treatment. The SMV thrombus has shrunk, the ascites has disappeared (yellow arrows), and the small bowel edema and mesenteric adipose tissue density are improved after treatment. Axial image (**a**). Coronal image (**b**)

**Fig. 3:**
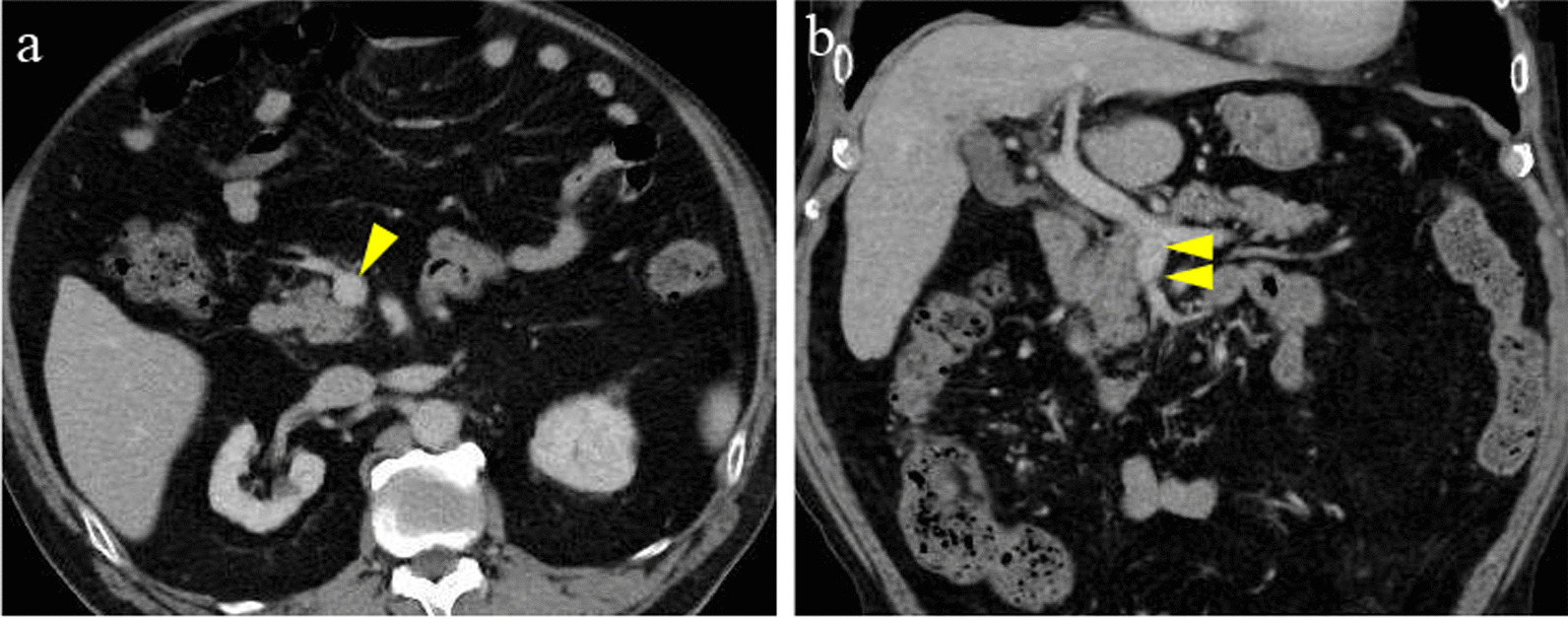
Abdominal computed tomography at 45 days after discharge. The SMV thrombosis has disappeared almost completely (yellow arrows). Axial image (**a**). Coronal image (**b**)

**Fig. 4:**
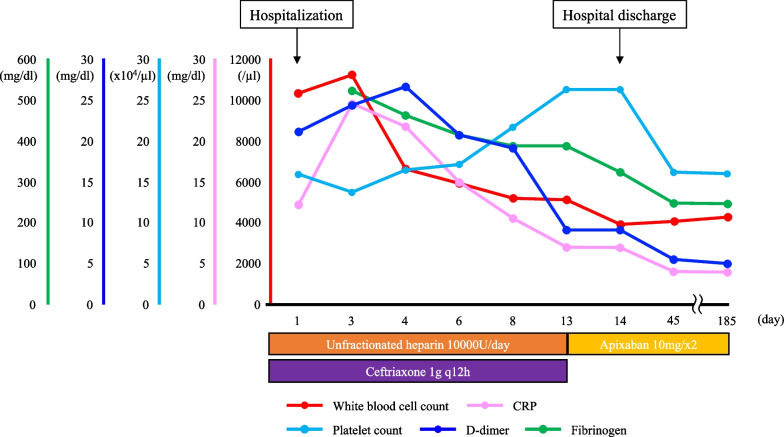
Overview of the patient’s clinical course over time. The diagram shows the levels of white blood cells (red), C-reactive protein (pink), fibrinogen (green), D-dimers (blue), and platelets (light blue) and treatment details from hospitalization to the most recent outpatient day available

The patient’s inflammatory and abdominal symptoms decreased gradually, and abdominal CT at 12 days after the start of treatment showed that the SMV thrombus had shrunk, ascites had disappeared, the small bowel edema had decreased, and mesenteric adipose tissue density had improved (Fig. 2). On hospital day 13, unfractionated heparin was changed to oral apixaban, and ceftriaxone was stopped. The patient was discharged the next day. Abdominal CT at 45 days after discharge showed almost complete disappearance of SMV thrombosis (Fig. 3). He has continued to take apixaban on an outpatient basis for 6 months and has had no recurrence of abdominal pain, and abdominal CT at 6 months after discharge showed maintaining disappearance of SMV thrombosis. Figure 4 shows our patient’s clinical course from hospitalization to the most recent outpatient day.

## Discussion

This case is an important report that suggests that among COVID-19 vaccines, mRNA vaccines may have caused venous thrombosis, particularly SVT. Mesenteric venous thrombosis is a type of SVT, that is, thrombosis that occurs in the mesenteric vessels of the venous circulation. Other types of venous thrombosis include deep vein thrombosis and pulmonary embolism, and the incidence of SVT is approximately 0.04% [2]. Mesenteric venous thrombosis is classified as acute, subacute, or chronic [3]. Acute mesenteric venous thrombosis is characterized by the sudden onset of abdominal pain, as in our patient, and is complicated by intestinal infarction in 30% of patients. When intestinal infarction occurs, the 30-day mortality rate is high (approximately 20%) [4].

Congenital risk factors for SVT include the JAK2V617F mutation, protein C deficiency, protein S deficiency, and antithrombin deficiency; acquired risk factors include cirrhosis, solid tumors, myeloproliferative neoplasms, inflammatory bowel disease, lipid syndrome, hormone therapy, and pregnancy [2]. The incidence of thrombosis due to malignant tumors varies greatly depending on the type of carcinoma and disease stage [6].

In our case, results of protein C, protein S, antithrombin, and other tests ruled out congenital and acquired risk factors for thrombosis. In addition, our patient had prostate cancer as a pre-existing condition, but because it was an early-stage cancer and had only recently been diagnosed, he had not received hormone therapy. Balabanova et al. reported that men with prostate cancer had a 50% increased risk of a first venous thromboembolism (VTE) in the 5 years following cancer diagnosis compared with men free of prostate cancer in the general population [5]. However prostate cancer reportedly has a lower incidence of cancer-related venous thromboembolism than other malignancies [6] and no association between prostate cancer and SVT has been reported. For these reasons, we thought it unlikely that prostate cancer was the cause of our patient’s SMV thrombosis.

Vaccines against COVID-19 have been developed by using two different technologies: viral vectors and mRNA [7]. Although mass vaccination worldwide has helped to prevent the spread of COVID-19, side effects of these vaccines are attracting attention. One particularly serious side effect of COVID-19 vaccines is vaccine-induced thrombotic thrombocytopenia (VITT) [7–9]. This complication is mainly associated with viral vector vaccines [7] and has an incidence rate of 1.5 to 3 per 100,000 vaccinations [7, 10]. Table 1 includes previous case reports of COVID-19 vaccine-induced splanchnic or mesenteric venous thrombosis [10–14], all of which have been due to viral vector vaccines. Additionally, in a systematic review by Zheng et al. on SVT following COVID-19 vaccination, only one case was caused by an mRNA-based vaccine [15]. Symptoms of VITT become apparent 5–30 days after vaccination [16]. VITT is most commonly associated with cerebral vein thrombosis, with central venous thrombosis accounting for 54% of cases, deep vein thrombosis for 36%, and SVT for 19%. Recent mechanistic studies have revealed that nucleic acids in the vaccine attach to PF4, triggering the formation of PF4-reactive autoantibodies and resulting in VITT. A similar mechanism has been postulated for heparin-induced thrombocytopenia [7, 8]. Cases of VITT have a high probability of positivity for anti-PF4 antibody, with a positive rate of 91%, but the causal relationship between thrombosis after mRNA vaccination and PF4 antibody is unclear and warrants further research [8, 17]. The primary treatment for VITT is non-heparin therapy, including corticosteroids, intravenous immunoglobulin, and platelet transfusion [7–9, 18], but mortality rates do not differ between patients who receive heparin therapy and those given non-heparin therapy [8].

**Table 1:** Reported cases of splanchnic or mesenteric venous thrombosis due to COVID-19 vaccination

No	Year/Author	Age	Gender	Pre-existing disease	Type of vaccination	Time from vaccination to onset	Anti-PF4 antibody	Treatment	Outcome
1	2022/Bogovic et al. [8]	40	Male	–	ChAdOx1 nCoV-19	10 days	Negative	IVIG, IVR, Operation	Alive
2	2021/Schultz et al. [12]	32	Male	Asthma	ChAdOx1 nCoV-19	7 days	Positive	LMWH, IVIG	Alive
3	2021/Scully et al. [12]	55	Female	–	ChAdOx1 nCoV-19	6 days	Unknown	–	Dead
4	2021/Scully et al. [13]	54	Male	–	ChAdOx1 nCoV-19	10 days	Negative	–	Dead
5	2021/Umbrello et al. [14]	36	Female	Asthma	ChAdOx1 nCoV-19	unknown	Positive	Unfractioned heparin	Alive
6	2021/Fanni et al. [15]	58	Male	–	ChAdOx1 nCoV-19	13 days	Unknown	–	Dead
7	Our case	80	Male	Hypertension, Hyperuricemia, Prostate cancer	mRNA-1273	6 days	Negative	Unfractioned heparin	Alive

To summarize the case, we report here: our patient received a dose of the mRNA1273 COVID-19 vaccine (Moderna) 6 days prior to onset of symptoms. Laboratory tests at diagnosis showed slightly decreased platelets and elevated D-dimer and fibrinogen levels, abdominal CT showed a thrombus in the SMV, and other risk factors for thrombus formation were ruled out. Testing for PF4 antibody was negative. The accumulated findings strongly implicated COVID-19 vaccine as the cause of our patient’s SMV thrombosis. His condition improved with conservative treatment comprising continuous intravenous infusion of undifferentiated heparin without catheterization or surgery. At 13 days after the start of treatment, our patient began to receive oral apixaban instead of intravenous heparin, and he has experienced no recurrence of symptoms.

## Conclusion

Here we report the first case of SMV thrombosis due to the mRNA-1273 vaccine. The possibility of venous thrombosis should be investigated in patients who present with abdominal pain soon after COVID-19 vaccination. Although the relationship between the COVID-19 mRNA vaccine and VITT is unknown, heparin therapy should be carefully performed, given the potential consequence of VITT.

## Data Availability

The datasets used and/or analyzed during the current study are available from the corresponding author on reasonable request.

## Abbreviations

mRNA: Messenger ribonucleic acid
PF4: Platelet factor 4
RT-PCR: Reverse transcription-polymerase chain reaction
SMV: Superior mesenteric vein
SVT: Splanchnic venous thrombosis
VTE: Venous thromboembolism
VITT: Vaccine-induced thrombotic thrombocytopenia
